# Orthopaedic Trainee Opinion on the Current Procedure-based Assessment Compared to its Predecessor: A UK Trauma Centre Perspective

**DOI:** 10.7759/cureus.7799

**Published:** 2020-04-23

**Authors:** Eamon Ramhamadany

**Affiliations:** 1 Orthopaedic Surgery, University Hospitals Coventry and Warwickshire National Health Service (NHS) Trust, Coventry, GBR

**Keywords:** training, orthopaedic, assessment, residency, procedure-based

## Abstract

Background

Procedure-based assessments (PBAs) were introduced as a formative assessment of surgical performance. UK trainees are currently expected to complete at least 20 PBAs each training year. A new PBA tool was introduced in August 2016 in order to address several criticisms with its predecessor. These included mandatory written feedback and increasing the number of global levels of assessment.

Objectives

Our study sought to identify the impact of these modifications to the PBA tool on its perceived utility as a formative assessment of surgical skill.

Study design & methods

Orthopaedic trainee registrars (ST3-ST8) holding a UK National Training Number (NTN) at a major UK trauma centre were invited to take part in the study. Each trainee completed an anonymous questionnaire that was designed to elicit the view of trainees towards the old and new PBA.

Results

Twelve trainees took part in the study. Most of them admitted receiving good quality feedback from their trainer using both tools (75% old PBA; 83% ew PBA). Most trainees (58%) felt that written feedback did not encourage verbal feedback from their trainer. Overall, trainees felt the new PBA global rating scale levels had made it a more accurate measure of surgical performance and allowed them to better appreciate the gradual improvement in their surgical skills throughout their training.

Conclusions

Fifty per cent of the trainees believed that the new PBA overall was a better formative assessment of surgical skill than its predecessor. A significant factor has been the introduction of a new global rating score rather than efforts to improve feedback. Further work should look to identify whether these views match those of other UK-based trainees on a larger scale.

## Introduction

Procedure-based assessments (PBAs) provide a formative assessment of surgical trainees in the UK [1]. Trainees are currently expected to complete at least 20 PBAs per year of training [2]. It was hoped that a PBA could be completed each time a surgical procedure was undertaken so that trainees could be provided with feedback and a global rating of their performance [3]. Increasing evidence has suggested that PBAs are being used as a summative tool of assessment [4-5]. Trainees are expected to achieve a Level 4 (able to perform procedure independently) in various surgical procedures in order to satisfy the Joint Committee on Surgical Training (JCST) requirement to complete their training [2]. It has been identified by the Darzi report that surgeons should be formally assessed throughout their training in response to previous surgical scandals such as the Bristol Heart Scandal [6]. The increased use of PBA as a summative tool is at odds with its original purpose as a formative assessment. As a result, there has been increasing frustration among trainees that PBAs are being used as a summative tick box exercise to satisfy the GMC and the deaneries [7].

There has a lot of criticism about the utility of the original PBA assessment tool. Thirty per cent of trainees commented that they failed to receive any feedback on their performance [5]. Saedon has identified feedback as the aspect trainees value the most from such assessments [8]. The inclusion of mandatory written feedback has been proposed as a way to address this shortcoming [5]. In response, the new PBA tool introduced in August 2016 has specified that written feedback is documented before an assessment can be validated by their trainer.

The current PBA tool has also sought to clarify the level of performance required to achieve each global rating by providing explanations alongside each. Khan has identified that the lack of explicitness as to the meaning of each level can lead to inaccuracies by assessors of work-based assessment [9]. Trainers may compare trainees against each other rather than against guidelines compromising criterion validity. Assessors may also fail to agree on the rating of a trainee, as they do not understand the level required to achieve each standard, which leads to reduced inter-rater reliability [10].

Williams has advised that between five and seven global ratings are required in the assessment to allow satisfactory construct validity [11]. The old global rating contained four levels, which may have been too few to distinguish between the levels of surgical expertise and subtle progression in trainees skills. The current PBA contains eight levels of performance expertise. Eardley comments that global scales that reflect increasing clinical sophistication are of greater utility to trainees [12]. It is hoped that the current PBA scale may be viewed by trainees as being a more accurate and dynamic way of determining performance level.

Research questions and hypothesis

Our study sought to identify the impact of the various modifications to the PBA tool on its perceived utility as a formative assessment of surgical skill. Van der Vleuten’s formula defines utility as relying on educational impact, validity, and reliability [13]. Increasing the global rating scale levels from 4 to 8 theoretically improves construct validity [11]. This should allow trainees to distinguish smaller steps in their surgical expertise throughout their training. Improving the understanding among trainees and trainers as to the performance required to achieve each global rating level should improve criterion validity. Furthermore, increased clarity should promote greater agreement amongst different trainers(inter-rater reliability) as to the correct global rating level of a trainee. It is hoped that the inclusion of mandatory written feedback should improve overall feedback quality and thus the educational impact of the PBA.

The research questions were as follows:

1. Do orthopaedic trainees view the new PBA as a more useful assessment of surgical skill?

2. Does the inclusion of mandatory written feedback improve feedback quality and thus educational impact?

3. Does the increase in the global rating levels provide a more accurate assessment of surgical skill expertise? (construct validity)

4. Does increased clarity of each global rating level’s meaning promote agreement amongst assessors in correctly identifying a trainee’s level of performance? (inter-rater reliability)

5. Does increased clarity of each global rating level’s meaning allow trainees and trainers to better understand the performance required to achieve each level? (criterion validity)

6. Are there any further factors or barriers that currently impact the utility of a PBA as a formative assessment tool?

## Materials and methods

Our study tests the hypothesis that trainees perceive the newer PBA to be an improved formative assessment tool by specifying written feedback, increasing the number of global rating levels and making the assessment tool easier to understand. These factors have been identified as shortcomings in the literature [8,10,14]. This lends our study design to a deductive approach that ascertains whether the new PBA is seen as better due to these factors.

Our project evaluated whether the modifications of the PBA have directly impacted on its perceived utility. We elicited the perspectives of trainees to both the original and new assessment tool to identify whether there has been a measurable improvement. A Likert attitude score can be used to quantify trainees' feelings towards the new and old PBA tools in order to try and elicit a measurable difference if it exists. Thomas states that an attitude Likert score assigns a value to the degree to which a participant agrees or disagrees with a certain value or statement [15]. We propose to use an attitude Likert scale as the basis for a questionnaire that will be used as the data gathering tool for the study.

Orthopaedic trainee registrars (ST3-ST8) holding a UK National Training Number (NTN) were invited to take part in the study. Each trainee was kindly asked to complete an anonymous questionnaire online within a two-month window. Ethical approval was sought and granted by the University of Birmingham, UK. Implied consent was assumed by a participant’s decision to voluntarily complete and submit their responses online.

A questionnaire was designed in order to elicit the views of trainees towards both the old and the current PBA. Participants were asked to clarify whether they had used and were familiar with both PBA tools. The subsequent questionnaire was initially divided into three parts, each looking at one modification to the assessment tool (feedback, global rating scale, clarity of the assessment). For each modification, trainees were asked to complete a five-point attitude Likert score to each statement for both the new and the old PBA. It was hoped that this could be used to test the impact of each modification of the assessment tool. Each attitude on the Likert scale could be assigned a value, 1=strongly disagree to 5= strongly agree, which would be used as quantitative data for the study. Participants were then asked whether they thought that, overall, the current PBA was a more useful tool of assessment than its predecessor. Trainees were then asked to rate the relative importance of each modification (written feedback, clarity, global rating) as well as verbal feedback on the success of a PBA. Free text boxes were then used to elicit qualitatively other factors that have improved as a result of the new PBA, any barriers to its success, and further ways in which the tool could be improved.

The results from the attitude Likert scores were quantitatively analysed using a student's t-test. Sullivan advises that although Likert scales can be viewed as ordinal data, parametric tests, e.g., the t-test, can be used if there are >5 observations and the data can be assumed to be normally distributed [16]. Furthermore, as the same sample population was used to compare trainees’ views before and after the introduction of the new PBA, a paired t-test was deemed to be most appropriate. The Statistical Package for the Social Sciences (SPSS; IBM Corp., Armonk, NY) was used to calculate a p-value for each modification, comparing the old with the new PBA. In order to compare the trainee's perceived relative importance of each modification to the success of a PBA, a one-way analysis of variance (ANOVA) was used with a Tukey-Kramer correction post hoc test. The ANOVA could be used to see whether there were any significant differences among the relative factors on PBA success and the Tukey-Kramer method would allow us to identify between which factors these were present. p<0.05 was accepted as statistically significant. The qualitative responses in the free-text boxes were reviewed to identify any common factors but no formal nVIVO analysis was performed.

## Results

Twelve out of 15 trainees (80%) contacted agreed to take part in the pilot study within the two-month period. Each participant completed an average of 0-2 PBAs per week using both the current PBA and its predecessor. Fifty per cent of trainees believed that the new PBA tool was a better formative assessment of surgical skill overall. The impact of each modification to the PBA tool was reviewed in turn and a p-value calculated for each attitude statement to look for a statistically significant improvement after the introduction of the new PBA.

The impact of mandatory written feedback is given in Table 1.

**Table 1:** Feedback with the current and original PBA tools Likert scale: 5 - strongly agree, 4 - agree, 3 - neutral, 2 - disagree, 1 - strongly disagree PBA: procedure-based assessment

Feedback questions	Old PBA					Old PBA		Current PBA					Current PBA		p-value old vs current PBA using t-test
	Attitude Likert					Mean	SD	Attitude Likert					Mean	SD	
	1	2	3	4	5			1	2	3	4	5			
I receive good quality feedback (n = participant)	0	1	2	8	1	3.75	0.75	0	1	1	10	0	3.75	0.62	1
Sufficient time for feedback	0	4	1	7	0	3.25	0.97	0	3	2	7	0	3.33	0.89	0.82
Feedback contributes to my surgical skills	0	1	4	7	0	3.5	0.67	0	1	3	8	0	3.58	0.67	0.76
	Attitude Likert						Mean Attitude Likert								
	1	2	3	4	5										
Written feedback in new PBA encourages verbal feedback (n=participant)	1	4	2	5	0		2.83								

Most trainees admitted receiving good quality feedback from their trainer using the old (9/12) and new (10/12) PBA assessment tool, as well as the feedback to positively develop their surgical skill (old PBA=58%, new PBA=66%). Only a minority of trainees admitted to their not being enough time dedicated towards feedback on the completion of the assessment (old PBA=33%, new PBA=25%). For each of the three statements on feedback, there was no statistically significant improvement following the introduction of the mandatory feedback specified in the new PBA tool (p>0.05). Interestingly, only 42% of recipients agreed that mandatory written feedback encourages better quality verbal feedback from the trainer. Two disgruntled trainees criticised the inclusion of written feedback as a barrier to the success of PBA. One stated that written feedback had made the tool ‘too long to complete’ timewise; another felt it had ‘worsened the experience of receiving feedback from the trainer’.

Table 2 shows the increase in the global rating scale levels from 4 to 8.

**Table 2:** Global rating scales using the current and original PBA tools Likert scale: 5 - strongly agree, 4 - agree, 3 - neutral, 2 - disagree, 1 - strongly disagree PBA: procedure-based assessment

Global rating scales	Old PBA					Old PBA		Current PBA					Current PBA		p-value old vs current PBA using t-test
	Attitude Likert					Mean	SD	Attitude Likert					Mean	SD	
	1	2	3	4	5			1	2	3	4	5			
Has enough levels to assess my performance	0	6	3	2	1	2.83	1.03	0	0	4	7	1	3.75	0.62	0.0086
Has helped me see the progression in my surgical skills	1	4	3	4	0	2.83	1.03	0	1	5	6	0	3.42	0.67	0.0024

There was a statistically significant improvement among trainees for both statements relating to the global rating scale following the introduction of the new PBA. There was a significant increase in the number of trainees who felt that an increase in the global rating scale levels had made it a more accurate measure of surgical performance (p=0.0086). They also felt that increasing the number of levels had allowed them to better appreciate the gradual improvement in their surgical skills throughout their training (p=0.0024). Trainees commented that ‘increased scales’ were a factor contributing to the overall success of the new PBA tool.

Table 3 shows the improvement in the clarity and understanding of the PBA tool.

**Table 3:** Clarity of the current vs the original PBA assessment tool Likert scale: 5 - strongly agree, 4 - agree, 3 - neutral, 2 - disagree, 1 - strongly disagree PBA: procedure-based assessment

Clarity of Assessment tool	Old PBA					Old PBA		Current PBA					Current PBA		p-value old vs current PBA using t-test
	Attitude Likert					Mean	SD	Attitude Likert					Mean	SD	
	1	2	3	4	5			1	2	3	4	5			
I fully understand what each global rating level means	0	3	4	4	1	3.25	0.97	0	3	2	6	1	3.42	1	0.166
My assessor understands what each global rating level means	0	6	2	4	0	2.83	0.94	1	3	3	5	0	3	1.04	0.338
there would be an agreement on the global rating level amongst different assessors	1	2	3	6	0	3.16	1.03	1	2	2	7	0	3.25	1.06	0.338

Most trainees felt unconvinced that their trainer understood what each of the global ratings meant with both the old (mean attitude score=2.8) and the new PBA tool (mean attitude score=3). One trainee commented that trainers would ‘ask where you should be for your year’ when assigning a mark. Two trainees felt that they had a better understanding of what each global rating meant after the introduction of the new PBA (5/12 old PBA vs 7/12 new PBA) (p=0.1661). A trainee commented that ‘there was still confusion over scoring levels’ with another uncertain as to why 4b was designed as a higher rating than 4a. One respondent stated that ‘increased understanding of the assessment process’ was required. Furthermore, there was no statistically significant improvement amongst trainees that the new PBA tool led to more agreement in their global rating from a variety of assessors (p=0.3388).

Table 4 shows the relative importance of factors affecting PBA assessment.

**Table 4:** Trainees perception of the most important factors of PBA assessment PBA: procedure-based assessment; ANOVA: analysis of variance

Overall importance to PBA assessment rank from 1 = least important to 5 = most important								
	1	2	3	4	5	Mean	SD	
Verbal feedback (n=participant)	1	1	0	1	9	4.33	1.37	
Written feedback	0	1	5	6	0	3.41	0.66	
Global rating scale	0	1	4	7	0	3.5	0.67	
Clarity of assessment (trainee/trainer understand it)	1	3	3	4	1	3.08	1.16	
ANOVA calculation								
Source	DF	Sum of Square	Mean Square	F Statistic	P-value			
Groups (between groups)	3	10.166663	3.388888	3.277166	0.0296704			
Error (within groups)	44	45.500004	1.034091					
Total	47	55.666668	1.184397					
Tukey-Kramer calculation								
Pair	Difference	SE	Q	Lower CI	Upper CI	Critical Mean	p-value	
Verbal feedback vs written	0.916666	0.293555	3.122643	-0.191784	2.025116	1.10845	0.136932	
Verbal feedback vs global rating	0.833333	0.293555	2.838767	-0.275117	1.941783	1.10845	0.200869	
Verbal feedback vs clarity	1.25	0.293555	4.258153	0.14155	2.35845	1.10845	0.021563	
Written feedback vs global rating	0.083333	0.293555	0.283876	-1.025117	1.191783	1.10845	0.997093	
Written feedback vs clarity	0.333334	0.293555	1.13551	-0.775116	1.441784	1.10845	0.852681	
Global rating vs clarity	0.416667	0.293555	1.419385	-0.691783	1.525117	1.10845	0.748082	

Verbal feedback from trainers was found to be the most important factor contributing to the success of PBA (mean score=4.3). A statistically significant one-way ANOVA (p=0.0297) was found. The Tukey-Kramer analysis found verbal feedback to be a statistically more important factor than clarity of the assessment tool (p=0.021). No statistically significant difference was found in the relative importance of factors among the other groups.

PBA as a lengthy tick box exercise

A common theme identified as a barrier to the success of PBA was the length of time required to complete the PBA assessment. Four out of nine free-text comments centred on there being a lack of time to complete the PBA assessment, with two criticising the ‘length of time required’. Three out of six comments for ways to improve the PBA further have suggested ways in which the tool can be shortened in length. These include ‘less free text’, ‘having just one box for feedback’ and ‘taking away mandatory feedback which makes the tool too long’. A further theme identified was the PBA tool being referred to as a tick box exercise. One trainee remarked that setting minimum numbers of PBA for annual review of competence progression (ARCP) have turned it into ‘a summative tick box exercise rather than a formative educational one’. A further respondent was more scathing, commenting that PBAs ‘were a paperwork exercise’ with ‘far too many tick boxes that nobody reads’. One suggested that the tick boxes included within the PBA tool be replaced by a more meaningful section such as ‘reflection’.

## Discussion

Feedback: written feedback does not improve the quality of feedback overall

Nearly all orthopaedic trainees in our study received good quality feedback from their trainer prior to the introduction of the current PBA. Previous studies have suggested 15%-30% of trainees fail to receive feedback on their performance following work-based assessments [5,14]. It may be the case that as trainees participating in the study were based at teaching hospitals, their assessors may be better trained at giving good quality feedback. Hence, the introduction of mandatory feedback into the new tool was not required. The trainees agreed that the feedback they received had a positive impact on their future surgical practice. Veloski has commented that feedback can change future clinical performance when provided by a ‘credible authoritative source’ [17]. Verbal feedback was identified as the most important factor in the success of a PBA. This agrees with previous studies analysing the effectiveness of PBA [8,18]. Specifying written feedback has been thought of as a way to encourage feedback quality and delivery [5,14]. The introduction of mandatory written feedback in the new PBA tool did not have a significant impact on trainee attitude towards feedback in our study. There is an ongoing debate in the literature as to whether written feedback supersedes verbal feedback. Elnicki and Veloski conclude that both verbal and written are equally effective feedback modalities, whereas Kluger felt verbal feedback to be inferior [17,19-20]. Our study suggests a preference for verbal feedback. One trainee suggested that inclusion of written feedback could worsen the quality of feedback received. Studies have shown that feedback quality has been affected by the trainer’s reluctance to record negative evaluations and skewed to positive/neutral comments [21-22]. One could hypothesise that written feedback could be inhibiting the trainer’s ability to be more critical about a trainee’s performance. Verbal feedback will be kept confidential between the trainee and trainer, whereas written feedback will be logged in the trainee’s portfolio and available for viewed by future assessment panels as part of annual appraisals.

Global rating scale - better criterion validity

Fifty per cent of trainees felt that the new PBA tool was a more useful formative assessment of surgical skill. The increase in the number of levels within the global rating was found to be the only factor identified in our hypothesis to show a statistically significant improvement in the assessment tool. Construct validity can be defined as the ability of a tool to distinguish between different levels of expertise [23]. The increase in the number of rating scales was viewed as a more accurate measure of surgical skill than the old PBA, which contained only four performance levels. Our study agrees with Williams that five to seven levels is ideal when designing a global rating scale for a work-based assessment [11]. Trainees also felt that the new scale within the PBA allowed them to see a gradual progression in surgical skill over time with each procedure. Eardley has shown that rating scales that reflect increased clinical sophistication have better construct validity and, therefore, the assessment is perceived as more useful [12]. This agrees with our hypothesis that improving validity contributes to better assessment utility as a product of Van der Vleuten’s framework [13].

Clarity of tool - an ongoing issue; elements still confusing

Our study suggests that the current PBA tool’s efforts to make its global rating easier to understand have not succeeded yet. It was hoped that the current assessment could improve criterion validity by providing a more explicit explanation as to what each rating level meant. Furthermore, this increased understanding would promote greater agreement amongst assessors as to how they rate a trainee’s performance (inter-rater reliability) [10]. Many trainees remain unconvinced that their trainer understands what each global rating level means. There could be two potential reasons for this. It may be that assessors need formal training in how to use a PBA assessment tool. Studies have shown that too few consultants have been formally trained in how to use these assessment tools [21,24]. Efforts to make the tool more explicit will, therefore, not be successful alone without efforts to ensure that assessors are adequately trained. However, most trainees did not feel that the old PBA tool possessed poor inter-rater reliability. Holmboe has shown that assessor training improves inter-rater reliability among trainers. The trainees in our study were based at a teaching hospital where it is likely that their assessors would have had formal training in the PBA process [25]. A more likely factor could be some trainers have not quite got to grips with the adjusted scoring levels. The trainees did comment that there was ‘confusion over new scoring levels’. Assessor familiarity has been shown to have an impact on workplace-based assessments (WBAs) [26].

Ongoing barriers to PBA success: time taken and perception as a tick box exercise

Our study has highlighted two important factors not accounted for by our hypothesis that have directly impacted the assessment’s perceived utility by trainees. Many of the trainees’ criticisms of the PBA tool was the time taken to complete the assessment. They complained about the ‘time it takes to complete’ due to the length of the assessment tool. Trainees’ claimed that ‘there were too many free text boxes for feedback’, ‘too many boxes which no one reads’ and ‘mandatory written feedback makes the tool too long’. Cross has previously stated that ‘time to complete’ PBA’s has compromised its utility [18]. Pereira claims that 82% of trainees have commented that the length of time taken to complete these work-based assessments have impacted negatively on training [27]. Time pressure was also reported as a major problem with WBA effectiveness according to Abdelaal [28]. Williams has suggested that assessment tools be kept as short as possible in order to reduce such a time burden [11]. Current evidence from our study suggests that the length of the current PBA tool is placing a negative impact on its utility and further efforts should be made to simplify the tool.

Our study also uncovered that many trainees still perceive PBAs to be a tick box exercise. One commented that the minimum numbers required for ARCP turn the PBA into a tick box summative exercise rather than a formative educational exercise. Studies have previously demonstrated that orthopaedic trainees view PBAs as a summative tick box exercise [4,29]. This has been re-inforced by Ali and Bindal who have stated that this has been driven by the desire to set minimum numbers of PBAs to be completed within each training year, which are then used summatively as part of the ARCP process [7,24]. Evidence suggests that WBAs are being increasingly misused despite being introduced as a formative assessment tool designed to encourage constructive feedback [4,28-29]. Trainees’ may also feel that PBAs are a tick box exercise due to a failure of engagement with the process of assessment. One trainee felt that the tool is ‘a paperwork exercise’ with ‘too many boxes’. Hunter has commented on a culture amongst trainees and trainers towards PBAs [5]. Trainees are more likely to benefit from such assessments when there is a culture of belief that such exercises are educationally beneficial [28]. A lack of understanding of the intended purpose of such assessment tools by the trainer could contribute to this negative sentiment [24,28-29]. Further effort should be made to ensure trainers and trainees are educated on the rationale behind such assessments. As Knowles states, adult learners need to understand the theory behind an educational tool if they are to be motivated to use it [30]. Furthermore, Van der Vleuten (1996) states that acceptability is a factor that contributes to an assessment’s utility [13]. The failure of trainees and trainers to believe in the purpose and benefit of PBA compromises this acceptability [28,29].

## Conclusions

In conclusion, most trainees believed that the new PBA is a better formative assessment of surgical skill than its predecessor. A significant improvement that has contributed to this sentiment appears to be secondary to a change to the global rating scale of performance. Mandatory written feedback and attempts to improve the clarity and understanding of the new tool have failed to have an impact. Trainees' issues with the length of the assessment tool and their perception of PBA as a tick box exercise shows that there are ongoing issues with acceptability, which have yet to be addressed.
